# Emerging Telehealth Advances for Mental and Cognitive Health in Late Life

**DOI:** 10.1093/geroni/igab046.2171

**Published:** 2021-12-17

**Authors:** Cindy Woolverton, Patricia Bamonti, Lauren Moo

## Abstract

Over the last year, mental health services offered virtually have increased significantly in response to COVID-19. The rapid adoption of telehealth practices has raised many questions about how to develop and deliver effective interventions for older adults targeting their mental and cognitive health. In this symposium, we present on the feasibility of mental and cognitive health interventions for older adults using telehealth methods with particular focus on how adoption of these telehealth tools have been impacted by the current pandemic. Dr. Touchett and colleagues will present data on the telehealth utilization disparities among older veterans with comorbid disabilities and discuss ethical considerations when providing care for older adults. Dr. Kornblith and colleagues will present pilot data on the feasibility of GOALS, a video telehealth cognitive remediation group intervention for older adults with cognitive and emotional dysfunction related to traumatic brain injuries. Dr. Gould and colleagues will present pilot data on the feasibility and preliminary efficacy of a brief video-delivered self-management intervention BREATHE for older veterans with anxiety disorders. Dr. Weiskittle and colleagues will present their work on the development and dissemination of a brief 8-week telephone group intervention for homebound older adults targeting social isolation. Dr. Jacobs and colleagues will share their findings of a telephone delivered mindfulness intervention for caregivers and persons with dementia. Finally, the discussant, Lauren Moo, MD, an expert in assessing the efficacy of telehealth interventions will tie findings together and provide directions for future research and innovation.

